# 13. ENDOCANNABINOID MODULATION OF DOPAMINE NEUROTRANSMISSION

**DOI:** 10.1093/schbul/sby014.048

**Published:** 2018-04-01

**Authors:** Michael Bloomfield

**Affiliations:** University College London

## Abstract

**Overall Abstract:** There are converging lines of evidence that the endocannabinoid system is involved in the pathophysiology of schizophrenia and that understanding these mechanisms may lead to novel treatment targets. In this symposium, we will present a series of experiments that link cannabinoid pharmacology to major fields of schizophrenia research including the dopaminergic, glutamatergic and serotonergic systems, glial cell function and the genetics of cognition.

Dopamine is a major neurotransmitter implicated in the pathophysiology of schizophrenia. Thus, understanding processes that modulate dopaminergic signalling may lead to new insights into the biology and treatment of this disorder. The endocannabinoids anandamide and 2-arachidonoylglicerol (2-AG) modulate dopaminergic neural activity through interactions with CB1 and CB2 receptors. CB1 antagonists inhibit the effects of drugs that potentiate dopaminergic activity, such as cocaine. There is also evidence of interactions between CB1 and CB2 receptors in terms of cannabinoid-mediated changes in dopaminergic function. We will present new evidence that CB2 receptor antagonism opposes the inhibitory effects of rimonabant on cocaine-induced hyperlocomotion. Thus, highlighting the co-modulatory role of CB1 and CB2 receptors on dopaminergic function.

We will then present a new study investigating the antipsychotic mode of action of cannabidiol (CBD). CBD attenuates the psychotomimetic effects of delta-9-tetrahydrocannabinol (THC) and there is evidence that CBD has antipsychotic effects in patients with psychosis. CBD prevents a range of behavioural impairments associated with the NMDA hypofunction model of psychosis measured in the pre-pulse inhibition, social interaction and novel object recognition tests following a two week exposure to the NMDA antagonist MK801. In addition, CBD, prevented neural (measured by delta-FosB) and microglia activation, and the decreased decrease in the number of medial prefrontal parvalbumin-positive neurons. The effects of CBD were blocked by pre-treatment with the 5-HT1A receptor antagonist WAY100635. This indicates that the antipsychotic effects of CBD may be mediated via 5HT1A-mediated mechanisms.

Next, we will describe the effects of CBD on glial cells. Glial cells, which express CB1 and CB2 receptors and synthesise endocannabinoid transmitters, have been implicated in schizophrenia whereby oligodendrocyte dysfunction has been associated with white matter deficits in the illness. In an investigation of the effects of CBD on a human oligodendrocyte culture (MO3.13), CBD administration resulted in diverse changes in the expression of proteins implicated in the pathophysiology of schizophrenia.

Finally, we provide further evidence that polymorphisms in cannabinoid receptor genes are associated with cognitive impairments in humans. In particular, the rs12720071 polymorphism T/T allele is associated with impaired working memory in patients with psychosis.

